# Analysis for genotyping Duffy blood group in inhabitants of Sudan, the Fourth Cataract of the Nile

**DOI:** 10.1186/1475-2875-11-115

**Published:** 2012-04-17

**Authors:** Agnieszka Kempińska-Podhorodecka, Oktawian Knap, Arleta Drozd, Mariusz Kaczmarczyk, Miroslaw Parafiniuk, Milosz Parczewski, Andrzej Ciechanowicz

**Affiliations:** 1 Medical Biology Laboratory, Pomeranian Medical University, Powstancow Wlkp 72, 70-111 Szczecin, Poland; 2 Independent Laboratory of Disaster Medicine, Pomeranian Medical University, Rybacka 1, 70-204 Szczecin, Poland; 3 Department of Biochemistry and Human Nutrition, Pomeranian Medical University, Broniewskiego 24, 71-460 Szczecin, Poland; 4 Department of Clinical and Molecular Biochemistry, Pomeranian Medical University, Powstancow Wlkp 72, 70-111 Szczecin, Poland; 5 Department of Forensic Medicine, Pomeranian Medical University, Powstancow Wlkp 72, 70-111 Szczecin, Poland; 6 Chair and Clinic of Infectious Diseases, Pomeranian Medical University, Arkońska 4, 71-455 Szczecin, Poland

**Keywords:** Duffy blood group, Malaria, Sudanese Arabs, *Shagia*, *Manasir*, SNP polymorphism, Real-Time PCR

## Abstract

**Background:**

Genetic polymophisms of the Duffy antigen receptor for the chemokines (DARC) gene successfully protected against blood stage infection by *Plasmodium vivax *infection. The Fy (a-, b-) phenotype is predominant among African populations, particularly those originating from West Africa, and it is rare among non-African populations. The aim of this study was to analyse the frequency of four Duffy blood groups based on SNPs (T-33C, G125A, G298A and C5411T) in two local tribes of Sudanese Arabs, the *Shagia *and *Manasir*, which are both from the region of the Fourth Nile cataract in Sudan.

**Methods:**

An analysis of polymorphisms was performed on 217 individuals (126 representatives of the *Shagia *tribe and 91 of the *Manasir)*. Real-time PCR and TaqMan Genotyping Assays were used to study the prevalence of alleles and genotypes.

**Results:**

The analysis of allelic and genotype frequency in the T-33C polymorphisms demonstrated a significant dominance of the *C *allele and *CC *genotype (OR = 0.53 [0.32-0.88]; p = 0.02) in both tribes. The G125A polymorphism is associated with phenotype Fy(a-, b-) and was identified in 83% of *Shagia *and 77% of *Manasir*. With regard to G298A polymorphisms, the genotype frequencies were different between the tribes (p = 0,002) and no single *AA *homozygote was found. Based on four SNPs examined, 20 combinations of genotypes for the *Shagia *and *Manasir *tribes were determined. The genotype *CC/AA/GG/CT *occurred most often in *Shagia *tribe (45.9%) but was rare in the *Manasir *tribe (6.6%) (p < 0.001 *Shagia *versus *Manasir*). The FY*A^ES ^allele was identified in both analysed tribes. The presence of individuals with the FY*A/FY*A genotype was demonstrated only in the *Shagia *tribe.

**Conclusion:**

This is probably the first report showing genotypically Duffy-negative people who carry both FY*B^ES ^and FY*A^ES^. The identification of the FY*A^ES ^allele in both tribes may be due to admixture of the non-African genetic background. Taken as a whole, allele and genotype frequencies between the *Shagia *and the *Manasir *were statistically different. However, the presence of individuals with the FY*A/FY*A genotype was demonstrated only in the *Shagia *tribe.

## Background

Africa is considered as the origin of modern humans and is one of the most ethnically and genetically diverse regions of the world [1]. The African populations were shaped by demographic forces that influence variation on a genome-wide scale, such as ancient migration events and fluctuations in population size, and by evolutionary forces that influence individual *loci*, such as natural selection and mutation [2]. A great impact on human evolution is associated with malaria, one of the most significant infectious diseases in the world [3]. Selection pressure by this pathogen on the human genome occurred at least 10,000 years ago. It has been generally recognized that malaria was one of the factors determining population increases in the incidence of atypical variants of haemoglobin, such as haemoglobin S (HbS), C (HBC) or E (HBE), as well as variability in the Duffy blood group system.

The Duffy antigen is located on the surface of red blood cells. Duffy antigens are coded on the Duffy antigen receptor for the chemokine gene (DARC), located on the long arm of chromosome 1 (1.q22-1.q23) [4]. The protein encoded by this gene is a glycosylated membrane protein, which is a non-specific receptor for several chemokines and can be utilized by human malarial parasites *Plasmodium vivax *and *Plasmodium knowlesi *in order to invade erythrocytes.

Genetic polymorphisms were identified in humans, which affect the expression of the Duffy antigen. Duffy antigen is present in two major allelic forms, differing in an amino acid at position 42 in coding sequence (G125A) (i.e. glycine in FY*A and aspartic acid in FY*B) [5-9] and point mutation at position T-33°C (previously described as T-46°C) in GATA box responsible for the FY*A^ES ^(ES = erythrocyte silent*) *and the FY*B^ES ^alleles (common African allele) [3,10-16]. These four alleles: FY*A, FY*B, FY*A^ES ^and FY*B^ES ^contribute to a total of ten genotypes: FY*A/FY*A, FY*A/FY*B, FY*A/FY*A^ES^, FY*A/FY*B^ES^, FY*B/FY*B, FY*B/FY*A^ES^, FY*B/FY*B^ES^, FY*A^ES^/FY*A^ES^, FY*A^ES^/FY*B^ES^, FY*B^ES^/FY*B^ES ^[17,18]. The last three genotypes of those above-mentioned are responsible for the phenotype Fy(a-, b-) which is responsible for resistance to malaria. This phenotype is predominant among African people, especially those originating in West Africa and it is rare among non-African populations [11]. This lack of DARC receptors among almost all individuals of West African origin has been suggested as a natural selection induced by *P. vivax *[19]. Moreover, a less common variation of the allele FY*X, associated with weak expression of *Fy^b ^*antigen was also identified [8,10,20].

Due to the characteristic distribution of DARC alleles: FY*A^ES ^and FY*B^ES^, they have been used to assess admixture and ethnic backgrounds of populations like Saudi Arabs [21], Israeli Jews [22,23], Grande Comore Island islanders (Njazidja) [24] and also some African descent populations [25]. Characterizing the pattern of genetic variation among ethnically diverse African populations is critical for reconstructing human evolutionary history, clarifying the population history of Africans and African-Americans and determining the proper design and interpretation of genetic disease association studies [26,27].

Genetic variation of many populations across Africa is enigmatic [28]. There are some very uncommon and unique African tribes that are not well genetically characterized. Current demographic and political situations in Africa make any anthropological research conducted in this area very valuable. The *Shagia *tribe, until recently, inhabited the area from the Kurti to the Fourth Cataract. This tribe led an agricultural lifestyle and was regarded as morally and culturally governed by the Sudanese Arabs. The village populations were not numerous (40-60 persons per village) and were located at small distances from each other in difficult to access areas. Residents were isolated geographically and culturally, which led to genetic isolation. Recently, the natural residence of this tribe has been changed due to the construction of dams in the Merowe at the height of the Fourth Cataract of the Nile river (Sudan), which required massive displacement and dispersion of the *Shagia *tribe. For that reason the presented study is exclusive and irreplaceable. The second tribe examined, the *Manasir*, lives approximately 80 km from the described Shagia villages and represents a population with admixture of other populations. Approximately 50,000-78,000 people (including the ethnically related *Shagia *and *Manasir *tribes) have been displaced to the settlements built in the Nubian Desert. Despite the undoubted benefits of the construction of new hydroelectric plants, such as universal access to electricity, there are far-reaching consequences for the environment and human health (e.g., the development of diseases, such as Snail fever).

The purpose of this study was to analyse the genetic structure of the *Shagia *and *Manasir *tribes using the variability of the single nucleotide polymorphisms in Duffy gene, such as two typical for African populations SNPs: T-33 C [5,29] and G125A [14,30], along with two other SNPs: G298A [31-33] and C5411T.

The distinction of the *Shagia *tribe is one of the reasons why the prevalence of gene polymorphisms in the FY (Duffy) is very interesting. The results obtained in this study, based on the biological samples collected before the tribe dislocation, may be helpful in determining the origin of this tribe and enriching the existing knowledge on population movements in the African continent.

## Methods

### Subjects

An analysis of polymorphisms was performed on 217 individuals (101 females and 116 males; median age 30 years). Genetic material (buccal swabs) was collected from 126 individuals of the *Shagia *tribe and from 91 representatives of the *Manasir *that consented to participate in the study. Both tribes inhabited the Fourth Cataract of the Nile at the time of the study.

The authors state their research conforms to the Helsinki Declaration and to local legislation. Informed consent from patients was a prerequisite. Institutional ethical clearance from the Ethic Committee of Pomeranian Medical University was obtained.

### Genotyping

Buccal swabs for subsequent DNA extraction were collected from all individuals that consented to participate in the study. Collected swabs were carefully dried in a separate area in order to avoid contamination. For DNA extraction, a BuccalAmp DNA Extraction Kit (Epicentre, Madison, USA) was used. Oligonucleotide primers and TaqMan probes for Duffy polymorphisms (i.e. Rs2814778, Rs12075, Rs13962 and Rs863002) were designed and synthesized by Applied Biosystems (ID: C__15769614_10, C__2493442_10, C__2493443_10, C__7480790_10, respectively). The fluorescence data were analysed with allelic discrimination 7500 Software, v.2.0.2.

### Statistical analysis

Statistical analyses were performed with the StatView^® ^program using the chi^2 ^Pearsons, Fisher test. Haplotype analysis using haplo.stats [34] package was made by R software, version 2.10.1. The validation of haplotypes and a linkage disequilibrium (LD) block of polymorphisms were performed using Haploview, version 4.02. *P *< 0 0.05 was considered statistically significant.

## Results

The identification of genes encoding the blood group antigens, such as DARC, has been used for many years to assess population admixture and ethnic backgrounds. Duffy polymorphisms are characterized for African populations, therefore identification of the *FyB^ES ^*variant in non-African populations is likely to be the result of admixture of the African-American genetic background [6,11,35]. The genotype and allele frequency distributions recorded for four analysed SNPs in two population groups, the *Shagia *and *Manasir *tribes, are presented in Table 1. Genotype distributions were consistent with the Hardy-Weinberg expectations for T-33 C and G125A SNPs only (Table 1).

**Table 1:** Allele and genotype association results for Duffy in the *Shagia *and Manassir

SNP	Allele/genotype	Allele frequency		OR (95% CI)	Fisher's Exact P-Value	Hardy-Weinberg's Exact *P*-Value	
					
		*Shagia*, n (%)	*Manasir*, n (%)			*Shagia*	*Manasir*
**T-33C**	***T/C***	**33/219 (13.1/86,9)**	**40/142 (22/78)**	0.53 (0.32-0.,88)	**0.02**		
	
	***TT ***	4 (3.2)	6 (6.6)		**0.01**	0.22	0.36
	***TC***	25 (19.8)	28 (30.8)				
	***CC***	97 (77)	57 (62.6)				

**G125A**	***G/A***	**235/17 (93,2/6,8)**	**169/13 (92.9/7.1)**	1.06 (0.5-2.25)	> 0.9		
	
	***GG***	3 (2.4)	1 (1.1)		0.58	**0.01**	0.37
	***GA ***	11 (8.7)	11 (12.1)				
	***AA***	112 (88.9)	79 (86.8)				

**G298A**	***G/A***	**162/90(64,3/35,7)**	**101/81 (55.5/44.5)**	1.44 (0.97-2.13)	0.07		
	
	***GG***	36 (28.6)	10 (11)		**0.002**	**< 0.00**	**< 0.00**
	***GA***	90 (71.4)	81 (89)				
	***AA***	0 (0)	0 (0)				

**C5411T**	***C/T***	**136/116 (54/46)**	**95/87 (52.2/47.8)**	1.07 (0.73-1.57)	0.8		
	
	***CC***	10 (7.9)	4 (4.4)		0.46	**< 0.00**	**< 0.00**
	***CT***	116 (92.1)	87 (95.6)				
	***TT***	0 (0)	0 (0)				

Pair-wise linkage disequilibrium (LD) between T-33 C and G125A polymorphisms was calculated using the normalized disequilibrium coefficient (D') and was equal 0.51 (Corr = 0,31, p = 5e^-10^).

The analysis of allelic and genotype frequency in the T-33 C polymorphisms demonstrated a significant dominance of the *C *allele and *CC *genotype (OR = 0.53 [0.32-0.88]) in both tribes. Moreover, the frequency of allele *C *was significantly higher in *Shagia *than *Manasir *(*p *= 0.02). The prevalence of *CC *genotype in the *Shagia *and the *Mansir *was respectively 77% and 62.6%. These results are in agreement with previous reports showing that the -33 C allele was present in 79% of South African blacks [25] and 78.3% of African-American populations [30]. Allelic and genotype frequencies in other analysed SNPs, i.e. G125A and C5411T were similar in both tribes and align with the data representative for Africans. The study showed that *AA *homozygotes are dominant in G125A polymorphisms among the members of *Shagia *and *Manasir *tribes (88.9% and 86.8%, respectively). With regard to G298A polymorphisms the genotype frequencies were different between the tribes (*p *= 0,002). It is worthy of note that no single *AA *homozygote was found in the examined groups although the presence of the *AA *genotype in sub-Saharan Africa was reported (0.99%). According to HapMap the *GA *heterozygotes among European populations predominate in G298A polymorphisms. On the other hand, the *GG *homozygotes, which were found in both analysed tribes, are typical of the Euro-Brazilians, Afro-Brazilians, Asian-Brazilians and Amerindians (*Cayapo/Yanomama*) [31].

The haplotype frequency was designated on the basis of two SNPs: T-33 C and G125A (Table 2). Duffy-negative blood groups were identified in 83% of the *Shagia *and 77% of *Manasir*, what is consistent with the epidemiological data which demonstrated that 70 to 80% of people in north-eastern Sudan had Duffy-negative blood group [36]. The frequency of the obtained haplotypes (Table 2) was compared to SNP genotypic data from HapMap Data Rel 27 and the similarity was observed between the examined groups and the population of African ancestry in the Southwest USA (ASW: CA = 0.81; TA = 0.07; TG = 0.12).

**Table 2:** Two-locus (T-33 C, G125A) haplotype analysis using HAPLO.SCORE, overall *p *= 0,08

Haplotype	Haplotype frequency		Overall haplotype frequency	HAP-Score	Overall P-Value
				
	*Shagia*	*Monassir*			
**CA**	0,83	0,77	0,80	-1,59	0,11

**CG**	0,04	0,01	0,03	-1,26	0,21

**TG**	0,29	0,06	0,04	1,36	0,17

**TA**	0,10	0,16	0,13	1,85	0,06

In the presented study, determination of Duffy genotype and phenotype was based on the T-33 C (GATA polymorphism) and G125A SNPs (Table 3) [10-12,15]. Twenty combinations of genotypes for the *Shagia *and *Manasir *tribes were examined (Table 3). There was a statistically significant difference in frequency of one of the genotype combinations, i.e. *CC/AA/GG/CT *(FY*B^ES^/FY*B^ES^) (*p *= 0.001) which occurred in 45.87% of the *Shagia *tribe but in only 6.59% of the *Manasir *tribe.

**Table 3:** Frequency of T-33 C, G125A, G298A and C5411T genotype combinations

Genotype combinations				*Shagia*, n (%)	*Manasir*, n (%)	Fisher's Exact P-Value	GATA genotype according T-33C	Deduced genotype according G125A	Predicted genotype	Predicted phenotype
**T-33C**	**G125A**	**G298A**	**C5411T**							

*TT*	*AA*	*GA*	*CT*	1 (0,8)	4 (4,4)	0,16	*GATA+*	FY*B/FY*B	FY*B/FY*B	Fy (a-, b+)

*TT*	*AA*	*GG*	*CT*	1 (0,8)	0 (0,0)	≥ 0,99	*GATA+*	FY*B/FY*B	FY*B/FY*B	Fy (a-, b+)

*TT*	*GA*	*GG*	*CT*	1 (0,8)	0 (0,0)	≥ 0,99	*GATA+*	FY*A/FY*B	FY*A/FY*B	Fy (a+, b+)

*TT*	*GA*	*GA*	*CT*	0 (0,0)	2 (2,2)	0,17	*GATA+*	FY*A/FY*B	FY*A/FY*B	Fy (a+, b+)

*TT*	*GG*	*GG*	*CT*	1 (0,8)	0 (0,0)	≥ 0,99	*GATA+*	FY*A/FY*A	FY*A/FY*A	Fy (a+, b-)

*TC*	*AA*	*GG*	*CC*	1 (0,8)	0 (0,0)	≥ 0,99	*GATA+/-*	FY*B/FY*B	FY*B/FY*B	Fy (a-, b+)

*TC*	*AA*	*GA*	*CT*	17 (13,5)	16 (17,6)	0,45	*GATA+/-*	FY*B/FY*B	FY*B/FY*B	Fy (a-, b+)

*TC*	*AA*	*GG*	*CT*	2 (1,6)	3 (3,3)	0,65	*GATA+/-*	FY*B/FY*B	FY*B/FY*B	Fy (a-, b+)

*TC*	*GA*	*GA*	*CT*	4 (3,2)	7 (7,7)	0,21	*GATA+/-*	FY*A/FY*B	FY*A^ES^/FY*B or FY*A/FY*B^ES^	Fy (a+, b-) or Fy (a-, b+)

*TC*	*GA*	*GG*	*CT*	1 (0,8)	1 (1,1)	≥ 0,99	*GATA+/-*	FY*A/FY*B	FY*A^ES^/FY*B or FY*A/FY*B^ES^	Fy (a+, b-) or Fy (a-, b+)

*TC*	*GG*	*GA*	*CT*	0 (0,0)	1 (1,1)	0,41	*GATA+/-*	FY*A/FY*A	FY*A/FY*A^ES ^or FY*A^ES^/FY*A	Fy (a+, b-)

*CC*	*AA*	*GG*	*CC*	4 (3,2)	0 (0,0)	0,14	*GATA-*	FY*B/FY*B	FY*B^ES^/FY*B^ES^	Fy (a-, b-)

*CC*	*AA*	*GA*	*CC*	2 (1,6)	4 (4,4)	0,24	*GATA-*	FY*B/FY*B	FY*B^ES^/FY*B^ES^	Fy (a-, b-)

*CC*	*AA*	*GG*	*CT*	20 (45,9)	6 (6,6)	**≤ 0,001**	*GATA-*	FY*B/FY*B	FY*B^ES^/FY*B^ES^	Fy (a-, b-)

*CC*	*AA*	*GA*	*CT*	64 (50,8)	46 (50,6)	≥ 0,99	*GATA-*	FY*B/FY*B	FY*B^ES^/FY*B^ES^	Fy (a-, b-)

*CC*	*GA*	*GG*	*CC*	2 (1,6)	0 (0,0)	0,51	*GATA-*	FY*A/FY*B	FY*A^ES^/FY*B^ES^	Fy (a-, b-)

*CC*	*GA*	*GG*	*CT*	2 (1,6)	0 (0,0)	0,51	*GATA-*	FY*A/FY*B	FY*A^ES^/FY*B^ES^	Fy (a-, b-)

*CC*	*GA*	*GA*	*CT*	1 (0,8)	1 (1,1)	≥ 0,99	*GATA-*	FY*A/FY*B	FY*A^ES^/FY*B^ES^	Fy (a-, b-)

*CC*	*GG*	*GG*	*CC*	1 (0,8)	0 (0,0)	≥ 0,99	*GATA-*	FY*A/FY*B	FY*A^ES^/FY*B^ES^	Fy (a-, b-)

*CC*	*GG*	*GA*	*CT*	1 (0,8)	0 (0,0)	≥ 0,99	*GATA-*	FY*A/FY*B	FY*A^ES^/FY*B^ES^	Fy (a-, b-)

For homozygotes with the inactive-GATA site and G125A genotypes: FY*B/FY*B or FY*A/FY*B, the deduced genotypes were FY*B^ES^/FY*BES and FY*A^ES^/FY*B^ES^, respectively and the phenotype was Fy(a-, b-).

The genotype and phenotype were consistent with G125A when the individuals were wild-type GATA site homozygous. According to Woolley *et al *[37] and Yazdanbakhsh *et al *[10], the mutation in the GATA box is associated with the functional allele FY*B [10,37]. However, Zimmerman *et al *[15] suggest that the T-33 C polymorphism affect *Fy *expression on the FY*A and FY*B alleles in a similar manner [15]. Therefore, in the case when an individual was characterized by heterozygous GATA site (GATA+/-) and the genotype FY*A/FY*B (for G125A), both types of genotypes, i.e. FY*A^ES^/FY*B or FY*A/FY*B^ES ^(phenotype Fy(a+, b-) or Fy(a-, b+)) can be predicted. Similarly, for FY*A/FY*A homozygous, two deduced genotypes such as FY*A^ES^/FY*A or FY*A/FY*A^ES ^(phenotype Fy(a+, b-)) were assigned. Moreover, Zimmerman *et al *[15] imply that the FY*A^ES ^allele is a new allele independent of African origin, as opposed to the FY*B^ES ^alleles predominating in African and African-American populations. The FY*B^ES ^allele was also *the most numerous *identified in the *Shagia *51 (+/- 2.5) and in the *Manarsir *32.5 (+/- 4).

The most remarkable finding in the presented study is the identification of occurrence of FY*A^ES ^allele in the analysed tribes, which is consistent with the anthropological data and may suggest free flow of the genetic material in the analysed populations, i.e. admixture of non-African population in some periods. The presence of individuals with the FY*A/FY*A genotype in the *Shagia *tribe, who are not found in the *Manasir *population (Table 3), indicates more numerous re-combinations among the *Shagia *than the *Manasir *people. Interestingly, admixture of non-African population in this geographically isolated tribe was additionally confirmed by the authors in previous studies [38]. Furthermore, the genetic structure of the *Shagia *population differs significantly from the *Manasir *population, where there was a free flow of genetic material as the *Manasir *tribe inhabits the region of the Fourth Nile Cataract with other populations such as the *Beniamer, Ababda, Foraui *and *Kesinger *tribes. It is worth noting that the FY*A^ES ^allele is not observed in a more ethnically diverse populations, for example, in some tribes from Papua New Guinea (i.e. South Wosera) [15].

Indigenous Africans are characterized by high levels of genetic diversity within and between populations [26]. Also, the mtDNA evidence has suggested that southern Africa and the remainder of sub-Saharan Africa were regionally differentiated [39]. It would be interesting to continue research using more powerful bio-markers, such as Y-chromosome and/or mtDNA. Unfortunately, both the nature of the research conducted by this study and the small amount and unique character of the collected material make it impossible.

## Conclusion

The present study demonstrated, by all accounts for the first time, Duffy-negative genotype combination i.e. FY*B^ES^/FY*A^ES^. Moreover, the results suggest the admixture of non-African genetic materials in both tribes. In the case of the *Manasir *population, it is in accordance with the collected ethnographic data, while with regard to the isolated *Shagia *tribe, which has not been anthropologically and genetically described so far, it is a useful trace for explaining the history of this population. The FY*A^ES ^allele, identified in both tribes, is not likely to be directly related to the exposure of a human genome to *P. vivax *but rather may be a consequence of admixture of the non-African genetic background.

## Competing interests

The authors declare that they have no competing interests.

## Authors' contributions

AKP and AC designed and implemented the survey and wrote the article. OMK and MP^5 ^were responsible for selection of the patients and collect of the samples. AKP, AD, MP^6 ^were responsible for genotyping. AKP and MK conducted the data analysis. All authors read and approved the final manuscript.
